# Lesions of the biliary system in Atlantic salmon (*Salmo salar* L.)

**DOI:** 10.1111/jfd.13724

**Published:** 2022-10-29

**Authors:** Lene R. Sveen, Elvis Chikwati, Alf Dalum, Trond M. Kortner, Jarle Ravndal, Olai Einen, Turid Mørkøre

**Affiliations:** ^1^ Norwegian Institute of Food Fisheries and Aquaculture Research (Nofima) Tromsø Norway; ^2^ Department of Paraclinical Sciences, Faculty of Veterinary Medicine Norwegian University of Life Sciences Ås Norway; ^3^ Cargill Aqua Nutrition Bergen Norway; ^4^ Cermaq Group AS Oslo Norway; ^5^ Department of Animal and Aquacultural Sciences, Faculty of Biosciences Norwegian University of Life Sciences Ås Norway

**Keywords:** Atlantic salmon, calcification, gallbladder, inflammation, pathology

Gallbladders with mucosal outpouchings (diverticula) were found in a population of Atlantic salmon farmed in sea water. Soft tissue calcification affected the skin, skeletal muscle and gut, as identified by histological observations. In addition, inflammation of muscle and intestinal tissue and lymphocytic and fibrous reaction were present in the liver. The general fish population was characterized by poor growth and unspecified mortalities. Here we describe the observed lesions and discuss them in the light of the general health status of the population.

## 1 STUDY POPULATION

This study was part of a feeding trial in Finnmark county, northern Norway. The fish used were 147 g Atlantic salmon smolts transferred to six commercial seawater cages in November 2018 (68,415 salmon per cage) and harvested in May 2020 (2.9 kg). The fish were fed according to nutritional requirements with either a constant or seasonal adjusted EPA + DHA regimes in triplicates.

In October 2019 (11 months at sea) and May 2020 (at harvest), fish were randomly selected from each of the sea cages (*n* = 10) for biometric determinations. Tissue samples from four fish per sea cage waere collected from pyloric caeca, distal intestine and liver (liver only from May sampling), while skin with attached red and white skeletal muscle was collected from five fish per sea cage. In May, gallbladders with visual pathological changes were collected from four individuals. Tissue samples were fixed in 10% phosphate‐buffered formalin and stained with haematoxylin and eosin (H&E) according to Li et al. (2019), Alcian blue and PAS (AB/PAS) according to Sveen et al. (2021) and Von Kossa according to Culling et al. (2014). For registered pathologies, the reader is referred to Appendix S1.

## 2 RESULTS

**FIGURE 1:**
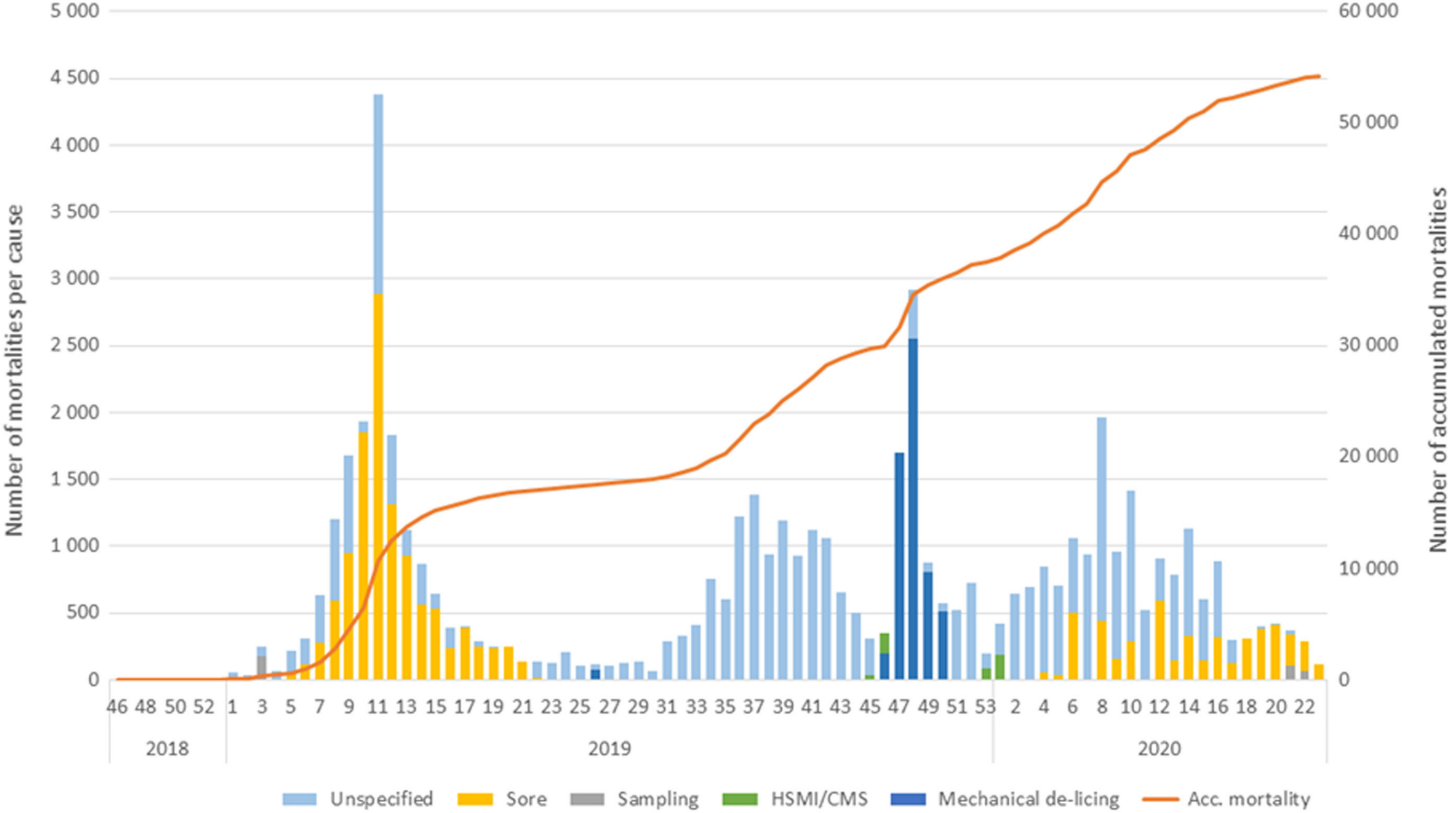
Weekly number of mortalities. Mortalities per cause (stacked bars) as recorded by the local fish health personnel and accumulated mortality (red line). Total initial farm inventory 410,490.

After 17.5 months in sea, growth was poorer than expected with 2.9 kg average harvest weight, whereas the expected slaughter weight for Atlantic salmon is 4–5 kg (Barrett et al., 2022). The cumulative mortality for the production was 13.3% with the weekly number of mortalities per cause given in Figure 1. Skin ulcers and unspecified mortalities were the main causes of losses, followed by mechanical delousing operations and some cases of heart and skeletal muscle inflammation (HSMI), with no dietary effects.

**FIGURE 2:**
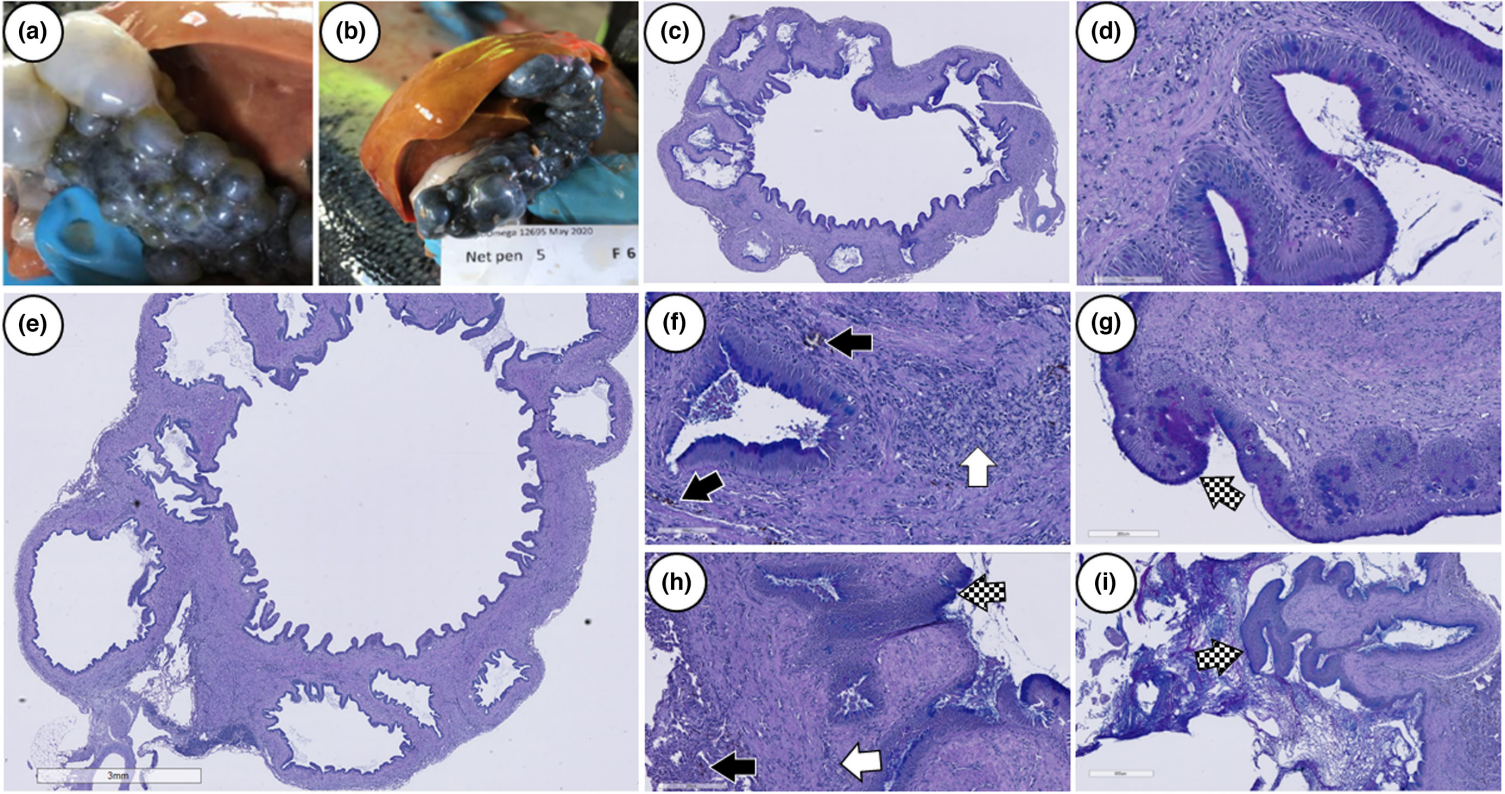
Pathology of the gall bladder. (a, b) Photos of gall bladders with lesions (c–i). Tissue sections of diverticula with characteristic connective tissue formation, eosinophilic granular cells (white arrow), melanized foci (black arrow), mucus (pattern arrow) response in the columnar epithelial tissue and lumen. Tissue sections stained with AB/PAS.

Gross pathology of the gall bladder ranged from minor to severe enlargement of the organ with numerous dark bulging lesions on the surface (Figure 2). The condition affected 4% of the sampled fish in October and 7% of the sampled fish in May. Microscopic findings showed diverticula of the mucosal wall, thickening of the fibromuscular layer, with the presence of eosinophilic granular cells and melanized foci (Figure 2f). An excessive mucus response was noted in the columnar epithelial tissue and lumen in some of the diverticula (Figure 2g–i).

**FIGURE 3:**
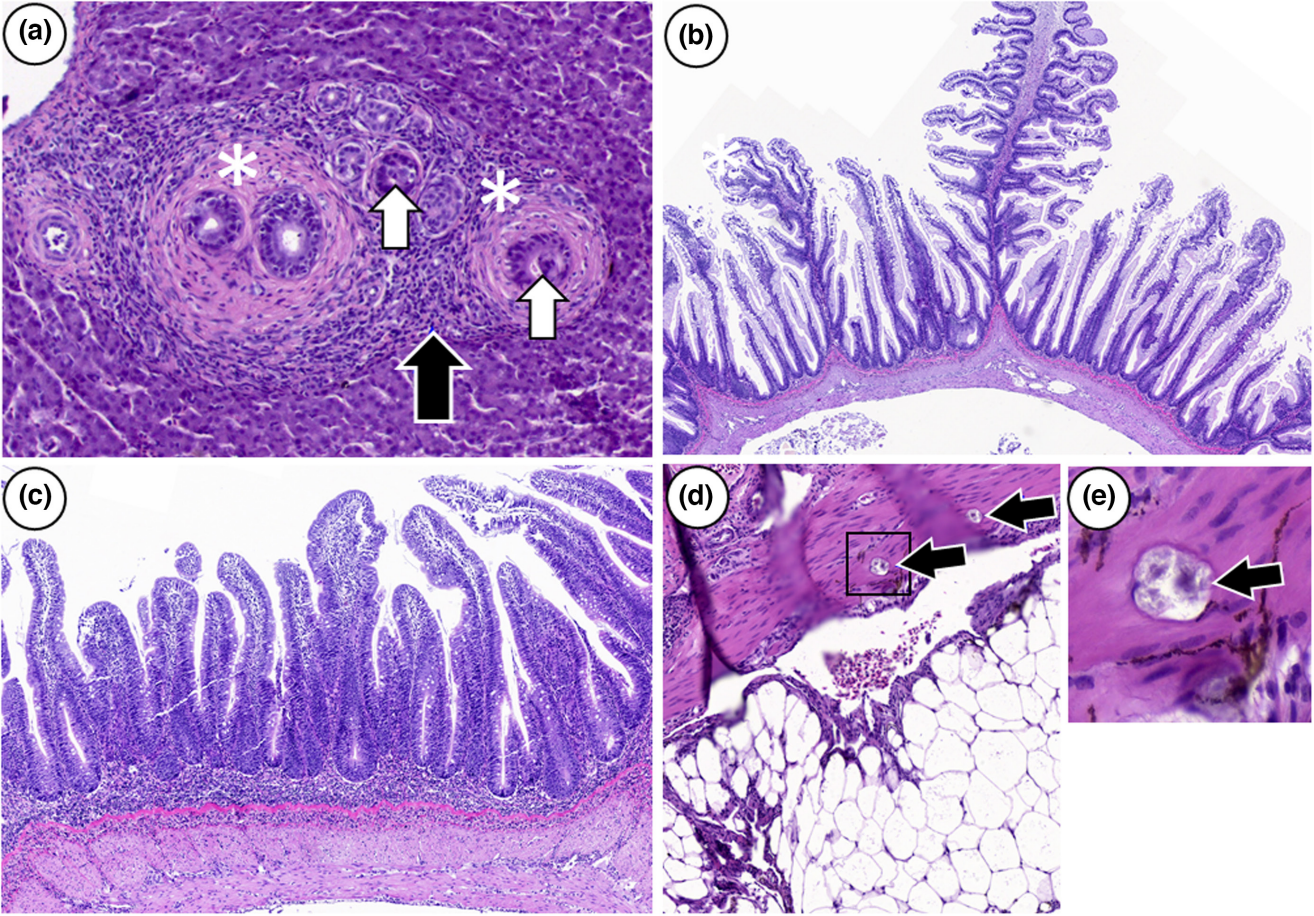
Lesions of the liver and distal intestine. (a) Peribiliary lymphocytic infiltration and fibrosis in the liver bile ducts (white asterisks), fibrous and lymphocytic infiltration (black arrow) and degeneration of the epithelium (white arrow). (b) Normal morphology of the distal intestine. (c) Moderate enteritis. (d) Multifocal lesions of soft tissue calcification in muscularis (black arrows), circular well‐defined calcified lesions accompanied by no tissue reaction (e) Magnified insert. Tissue sections stained with H&E.

Mild to severe lipid accumulation (steatosis) was observed within the enterocytes in pyloric caeca affecting 92% of the samples in October 2019 and 38% in May 2020. In the distal intestine, 70% of the samples had mild to marked inflammation in October, whereas in May, 50% of the samples were characterized by infiltration of the submucosa and lamina propria by a mixed population of inflammatory cells, shortening of mucosal folds and loss of supranuclear vacuoles in the enterocytes (Figure 3). In the liver, a peribiliary lymphocytic and fibrous reaction was observed as the main pathological finding (Figure 3), affecting 46% of the samples at harvest.

**FIGURE 4:**
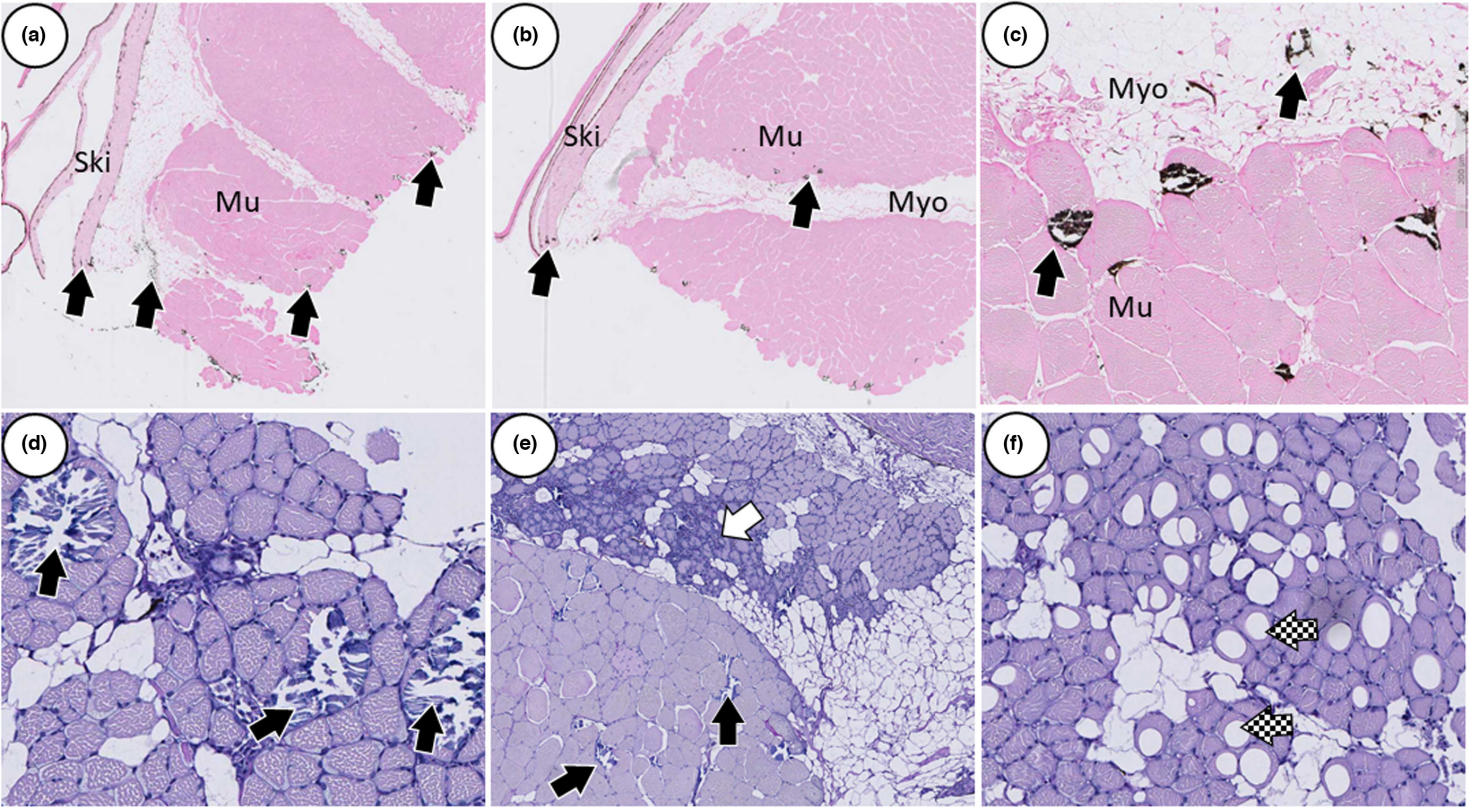
Calcification of skin and muscle tissue. (a–c) Calcified lesions (black arrow) were found in skin (Ski) dermis, myosepta (Myo), red and white muscle tissue (Mu). (d–f) Red and white muscle tissue with inflammation (white arrow) and vacuolization (pattern filled arrow). Tissue section (a–c) stained with Von Kossa, (d–f) with AB/PAS

Mineralized foci were present in the skin, skeletal muscle, pyloric caeca and the distal intestine. In October 2018, 73% of the skin dermis‐, myosepta‐ and muscle tissue were affected, accompanied by inflammation and vacuolization of red muscle tissue (Figure 4). In May 2020, 70% of the skin samples, 38% of distal intestine and 8% of the pyloric caeca samples had calcified lesions (Figures 3 and 4), while only one fish had inflammation in red muscle tissue.

## 3 DISCUSSION

**FIGURE 5:**
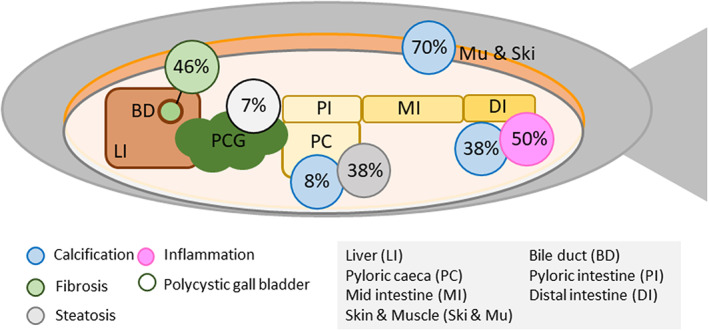
Pathological findings at harvest. The percentage of lesions is given as (*n* of lesions/*n* of samples analysed) separately for each tissue. The percentage of nodular gall bladders is given by (*n* observations/total number of fish sampled at harvest).

Here we present findings from a population of A. salmon, which upon harvest was characterized by poor growth, unspecified mortalities, incidences of calcinosis and lesions of the digestive and biliary system (Figure 5). The recorded causes of mortality and the average mortality rate represent the general trend in the industry (Sommerset et al., 2020). The large size of the diverticula suggests that this condition must have persisted for some time. The nature of the calcified foci and fibrosis of the hepatic duct further suggests a chronic state of the lesions. Such a chronic disease progression likely reduces the fitness of the animals and overlaps well in time with the unspecified mortalities recorded for this production. The high incidences of lesions in the gut and the biliary system likely affected nutrient digestion and absorption negatively, which may have contributed to the poor growth observed for the population.

The lesions of the gallbladders resemble that of intramural diverticulosis described in humans (Mark & Melnick, 1964; Ross et al., 1955). Ross et al. describe these diverticula as a likely consequence of chronic inflammation and increased luminar pressure, resulting in pulsion diverticula into the loose stroma of the gallbladder wall. Here, the high percentage of lesions in the biliary system and gut may indicate that these events are connected. Bile acids are further vital for the absorption of dietary fat and fat‐soluble vitamins such as Vitamin D. Dysregulation of vitamin D is related to calcification processes (Black & Kanat, 1985); hence, it is plausible that the biliary lesions may be connected to the calcified foci. Unfortunately, liver/gut and skin/gallbladder samples were collected from different individuals, and this hypothesis needs further elucidation.

The samples for this study were collected from random fish at a commercial production site; hence, we believe that the findings are representative for the population. The series of lesions may represent a health problem which may have been overlooked previously.

## FUNDING INFORMATION

This work was funded by the project ‘Omega‐3 for salmon under Arctic conditions – optimization of use and effects on fish health and fillet quality’ which was supported by Researcsh Council of Norway grant no 296296 – RFF ARKTIS and the industrial partners Cermaq and Cargill.

## CONFLICT OF INTEREST

The authors declare that there are no conflicts of interest associated with this publication.

## Supporting information


Appendix S1

## Data Availability

The data that support the findings of this study are available on request from the corresponding author. The data are not publicly available due to privacy or ethical restrictions.
